# Activation of Liver Cirrhosis: Control by Lymphosorbtion

**DOI:** 10.1155/1995/25076

**Published:** 1995

**Authors:** Teimuraz L. Pirtzchalava, Dmitry A. Granov, Pavel G. Tarazov

**Affiliations:** Department of Surgery (1,2) and Division of Angio/Interventional St. Petersburg Research Institute of Roentgenology and Radiation Therapy St. Petersburg Russia


*HPB Surgery,* 1995, Vol. 9, pp. 57
Reprints available directly from the publisher
Photocopying permitted by license only

© 1995 OPA (Overseas Publishers Association)
Amsterdam B.V. Published in The Netherlands
by Harwood Academic Publishers GmbH
Printed in Malaysia

### Activation of Liver Cirrhosis: Control by Lymphosorbtion

TEIMURAZ L. PIRTZCHALAVA, DMITRY A. GRANOV, PAVEL G. TARAZOV

Department of Surgery (1,2) and Division of Angio/Interventional, St. Petersburg Research Institute of Roentgenology and Radiation Therapy, St. Petersburg, Russia

*(Received April 12, 1995)*

**LETTER TO THE EDITOR**

EDITOR — Activation of liver cirrhosis (hepatocellular insufficiency, increase of ascites and gastroesophageal varices, worsening of encephalopathy) is very difficult to cure. Conservative management is often ineffective, and many patients die during the episode of activation. We report our experience with control of this syndrome by lymphosorbtion.

Combined treatment, lymphosorbtion plus medical therapy, was accomplished in 28 patients with Child-Pugh's Class C liver cirrhosis (Group 1). Lymph purification and reinfusion (500 ml to 1500 ml daily) was performed using chronic external surgical catheterization of the thoracic duct. The carbon adsorbent with fibers of $8 \times 10^{-3}$ mm to $12 \times 10^{-3}$ mm diameter and $2m^2/g$ external geometric surface was used for lymph purification. Control prospective Group 2 of 30 patients with C Class cirrhosis received only medical therapy.

Control of activation was achieved in 25 (89%) vs 17 (56%) patients,respectively. Hospital mortality rates were 4% and 17% in Groups 1 and 2. In two weeks after beginning of the treatment,decrease of ascites was seen in 100% and 70% of patients, respectively. Gastroesophageal varices decreased in diameter in 50% vs 0%, and encephalopathy regressed in 90% vs 30% of patients. The duration of hospital stay was shorter by two times in Group 1 than in Group 2.

These data show that lymphosorbtion combined with medical therapy is more effective than medical therapy alone in the management of patients with liver cirrhosis complicated by activation.

Teimuraz L. Pirtzchalava, MD
Dmitry A, Granov, MD
Pavel G. Tarazov, MD
St. Petersburg Research Institute of
Roentgenology and Radiation Therapy,
Pesochny-2, St. Petersburg, 189646, Russia

